# The dynamics of decision-making and action during active sampling

**DOI:** 10.1038/s41598-021-02595-3

**Published:** 2021-11-29

**Authors:** Duygu Ozbagci, Ruben Moreno-Bote, Salvador Soto-Faraco

**Affiliations:** 1 Center for Brain and Cognition and Department of Information and Communications Technologies, Pompeu Fabra University, Barcelona, Spain; 2 Institut Català de Recerca i Estudis Avançats (ICREA), Barcelona, Spain

**Keywords:** Decision, Decision, Human behaviour

## Abstract

Embodied Cognition Theories (ECTs) of decision-making propose that the decision process pervades the execution of choice actions and manifests itself in these actions. Decision-making scenarios where actions not only express the choice but also help sample information can provide a valuable, ecologically relevant model for this framework. We present a study to address this paradigmatic situation in humans. Subjects categorized (2AFC task) a central object image, blurred to different extents, by moving a cursor toward the left or right of the display. Upward cursor movements reduced the image blur and could be used to sample information. Thus, actions for decision and actions for sampling were orthogonal to each other. We analyzed response trajectories to test whether information-sampling movements co-occurred with the ongoing decision process. Trajectories were bimodally distributed, with one kind being direct towards one response option (non-sampling), and the other kind containing an initial upward component before veering off towards an option (sampling). This implies that there was an initial decision at the early stage of a trial, whether to sample information or not. Importantly, in sampling trials trajectories were not purely upward, but rather had a significant horizontal deviation early on. This result suggests that movements to sample information exhibit an online interaction with the decision process, therefore supporting the prediction of the ECTs under ecologically relevant constrains.

## Introduction

The classical view of decision-making was founded on the idea that action is executed after a decision has been made, in a serial fashion^1,2^. This idea assumes a temporal and functional separation between the decision-making processes and the ensuing motor processes that implement that decision. Recent behavioural studies have challenged this strictly serial view and proposed, instead, that the choice execution process may begin before the decision process has concluded, de facto introducing the parallel view of decision-making^3,4^. This parallel view states that there is an ongoing information flow from decision to action systems well before the decision process has been fully completed. According to this view, not only decision and action may coexist, but choice movements may be updated online based on newly acquired evidence^5^.

To investigate the putative interaction between action and decision as it unfolds in time, some studies have used decision-making tasks which require continuous control of action. These tasks track responses executed on devices like joysticks, robotic handles, computer mice, or freely with hand reaching movements^6–9^. Since these responses have a wide temporal and spatial span, they make it possible to study, and compare the movement dynamics during the decision-making process.

A typical finding that emerges from continuous movement paradigms when subjects must move toward one out of two alternative targets, is the prevalence of movement trajectories that are not perfectly direct to the chosen target^9^. These findings have shown that the initial phase of the response movement weighs in the paths to the two possible targets, maintaining a compromise which is later resolved by diversion of the trajectory committing to one of the targets^10,11^. Some scholars attributed these averaged movements to an error in movement planning or to uncertainty of the movement goals^12,13^. However, in decision making literature these averaged movement trajectories are commonly interpreted as a case of movement being planned and executed online during the decision process and more importantly, that there is a continuous crosstalk between these two processes^14,15^. An exacerbated expression of this online crosstalk are changes of mind, trials in which the subject’s response movement starts off toward one target but corrects on-the-fly toward the alternative target^7^. In general, these findings motivated the parallel view of decision-making, which focuses on the ongoing one-way flow of information from decision to action.

Although the parallel view of decision-making assumes a richer interaction between action and decision than the strictly sequential view, it only accounts for the forward influence from decision to action. However, there is evidence for backward influence from action on decision as well. For example, Burk, and colleagues (see^7^) showed that when the spatial distance between two response options is large, subjects make less changes of mind than when the distance between targets is shorter. This means that action costs are considered and influence the outcome of the decision process. In a similar vein, Cos and colleagues (see^16^) found that the amount of effort required to perform the response action biased performance in a decision-making task. There is, still, another type of backward influence from motor to decision processes: when actions help accrue information relevant for the decision. The present study addresses precisely this case.

We can frame the evidence mentioned above under Embodied Cognition Theories (ECT) of decision-making, whose common characteristic is the influence of action dynamics on decision as well as the influence of decision on action. Indeed, drawing connections between motor processes and decision-making has a conceptual grounding on the wider framework of sensorimotor and embodied views in cognitive sciences^17–19^, a general conceptual shift that has pervaded recent views in decision-making. One clear example is Lepora & Pezzulo’s Embodied Choice Model^20^. The model proposes a two-way online interaction between motor actions and decision processes and that this interaction allows for a fast update of movement and decision processes. A typical argument by example often used to support this view is that, in nature, animals must move about (their body and/or sensory epithelia) to be able to gather information that is relevant to making subsequent choices and planning upcoming actions^20^. To use the information gained through movement though, there needs to be a backward flow of information from action-related motor processes to decision-making.

**Figure 1:**
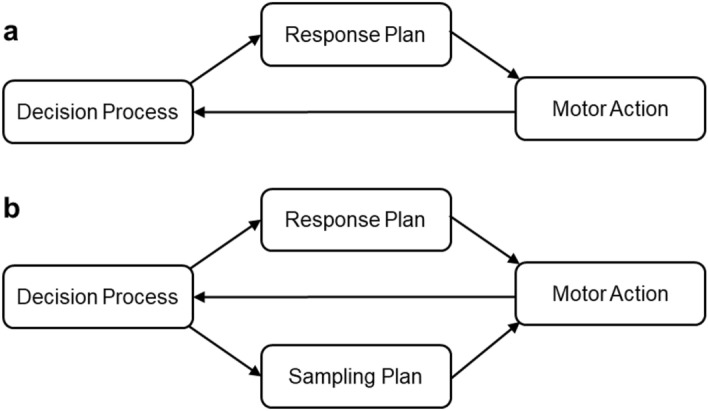
Interactions between motor action and decision in tasks without (**a**) and with (**b**) active information sampling. (**a**) In majority of the decision-making tasks decision process feeds the response plan which gets executed with a motor action. While the action continues, the output of the action feeds back into the decision process. This is not a fully embodied scenario, since actions do not bring an information change. (**b**) In a fully embodied scenario considered here, two different action plans, for sampling and for responding, are allowed to unfold in parallel. The decision process has a feedforward influence on motor output, whereas sampling influences decision via feedback from the motor action. In contrast to panel (**a**), the executed motor action implements both responding and sampling of information.

Despite the logical emphasis that embodied views make on information sampling movements, this notion has not been implemented in experimental tasks to support the ECTs. In fact, in most of these decision-making tasks, the stimulus information is available all at once and static, without any dependency upon the participant’s movement^8,20–22^. The interactions which can be potentially at play in these types of tasks have been illustrated in Fig. 1a. Because the actions performed to report a choice are inconsequential to the inflow of information used to reach that decision, these tasks cannot capture all possible interactions between action and decision proposed by ECTs. Therefore, there is a need for tasks that can reveal the two relevant aspects of actions to identify the potential interplay between motor and decision processes. This interplay, which has motivated the task used here to test decision-making under ECTs, is illustrated in Fig. 1b. Here, we assume that there are two types of action plans which are critical in an embodied decision-making scenario, the ones necessary for response itself, and the ones necessary for information sampling. Both of them interact with the decision process, and mediate both feed-forward and feedback interactions.

In conclusion, we believe that the generality of the interplay between decision and action, and by proxy, of the embodied decision framework, have not yet been tested in all its critical components. In the present study, we aim to testing the ECTs’ predictions with a task in which information accrual depends on the subject’s actions. Empirical evidence regarding such a scenario is still scarce. We have developed a novel mouse-tracking task in which action is necessary both to sample information and to indicate the decision. To be able to single out one from the other, movements directed at sampling information and movements to execute the response have been made orthogonal. That is, it is possible for the subjects to accumulate all the information first and then make the choice, to make a choice at once without any accumulation of information, or to do anything in between. Since trials have a time limit, the orchestration among information sampling actions and choice actions becomes strategic. Although sampling and response actions have orthogonal axis, one critical aspect of the task is that both action plans are executed via the same effector, so that the final motor output must synthesise the two plans if they are to co-occur, as the theory predicts. Similar to other mouse-tracking studies, the main test of our hypothesis depended on the analysis of metrics obtained from the trajectories^23^.

Our hypothesis, derived from the ECTs^14,20^, is that the actions related to the decision-making process and the actions related to information sampling used to reach that decision are subject to significant online interaction. We first show that, in our task, trajectories depend on the amount of available information such that participants move to sample information when needed. Second, we demonstrate that the decision-making process transpires even at the initial phases of the information sampling movements, so that trajectories are biased towards one (usually the chosen) target much before all the information has been gathered. These results do not only suggest that the decision-making process pervades information sampling actions, but also that decision, actions and information sampling may be orchestrated in parallel, and not necessarily in a strictly sequential fashion.

## Methods

### Participants

Twenty-one voluntary participants joined the experiment (12 women, 9 men, average age 23.5 years). Participants were recruited from the database of the Center for Brain & Cognition (University Pompeu Fabra) and were paid 10 euros per hour in exchange for their participation. They were all right-handed and had normal or corrected to normal vision with no reported history of motor problems related to the upper limbs. Before proceeding with the experiment, all subjects read and signed an informed consent form. The research was conducted in accordance with the Declaration of Helsinki, institutional guidelines and regulations. The experimental protocol was approved by the ethics committee CEIC Parc de Salut Mar, Universitat Pompeu Fabra. Before conducting the hypothesis-driven data analyses, we excluded data from two subjects whose accuracy was below 75%. This ensured sufficient number of correct trials for obtaining reliable trajectory averages.

### Experimental setup

Participants were asked to perform a visual object categorization between “edible” vs “non-edible” in a two-alternative forced choice (2AFC) paradigm. We used 63 edible and 63 non-edible object images from the Amsterdam Library of Object Images^24^, and each of them was presented only once to each participant, obtaining a total of 126 different trials per participant. To control for possible effects of colour cues, we used, achromatic versions of the images. Stimulus display and the task were programmed with MATLAB, PsychToolBox^25^. Visual stimuli were presented on a Cambridge Research Systems, Display +  + monitor (1920 × 1080 pixels, 32’’, 100 Hz refresh rate). Responses were recorded through a computer mouse (HP USB Optical Scroll Mouse), and the cursor location was recorded at 100 Hz (at every display refresh frame). The participant’s task involved moving the cursor from a home position at the bottom centre of the display to the right or left response areas, depending on the choice regarding the image presented at the top centre (locations and other details are described below).

For each subject, the total of 126 trials were divided, randomly and equiprobably into three different movement-to-visibility conditions: No Blur (NB), Low Blur (LB) and High Blur (HB). In the NB condition, the images were fully visible (without any blur) from the beginning of the trial, and therefore visibility was not contingent on action. For the other two conditions, in order to implement movement-dependent updating of information, we manipulated the visibility of the object images as a function of mouse position. We used a dynamic filter mask over the image to blur the image. The filter convolved each pixel with the neighbouring pixels with a Gaussian kernel with standard deviation (sd) proportional to the vertical distance between current cursor position and the target image at the top centre of the display, denoted *d*_*v*_ (measured in pixels). In the LB condition, the Gaussian mask had sd = *d*_*v*_/120, whereas in the HB condition the Gaussian mask had sd = *d*_*v*_/60. This effectively made blur (hence, image visibility) depend on the participants’ movement, so that moving upward de-blurred the target image (i.e., the shorter the vertical distance to target, the smaller *d*_*v*_, and hence the lower the sd and the higher the visibility). The difference between the two blur conditions was the gain in visibility as a function of distance.

### Procedure

**Figure 2:**
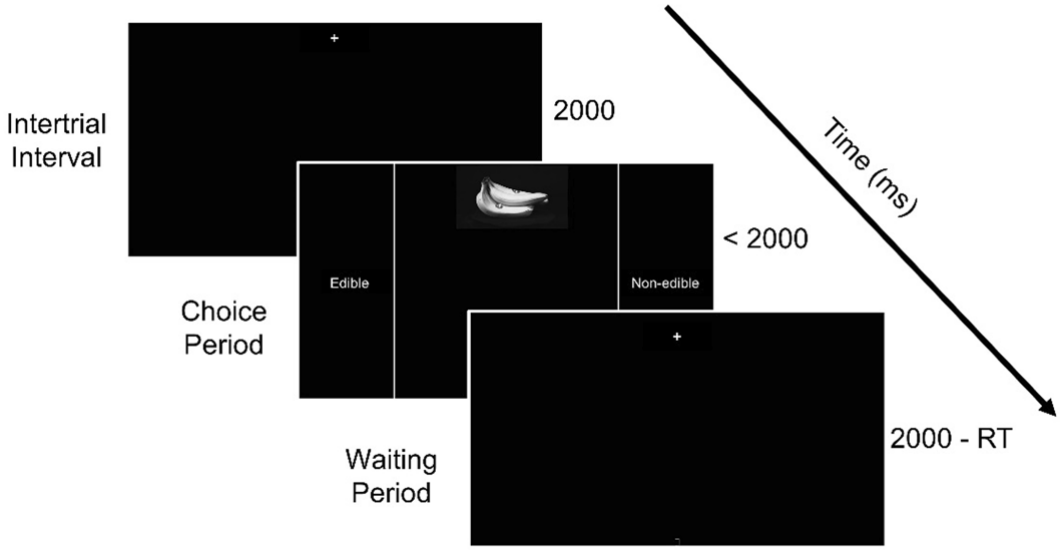
Schematic illustration of a trial sequence. Each trial was preceded by a 2000 ms inter-trial interval displaying a fixation cross. Then, the stimulus and the choices were presented on the screen until response, with a deadline of 2000 ms. Response areas, left and right of the display, are denoted by straight vertical lines. All trials were equated to the same duration, 2000 ms by adding a waiting time if necessary. *RT* reaction time.

Each subject completed the task in a darkened, sound-attenuated laboratory room. Subjects completed a training session prior to the experimental block. The training consisted of 18 trials (6 from each blur condition in a random order) in which we used novel images that did not appear in the experiment. Before each trial started, the subject moved the mouse cursor to the bottom-centre home area (height = 10 × width = 15 pixels, centre x, y coordinates: 960,1075 pixels). The trial began with the image (265 × 192 pixels) appearing at the top-centre of the monitor (x-coordinates: 827 to 1092, y-coordinates: 0 to 192 pixels). As soon as the image appeared, the subject was free to move the mouse to indicate her choice by reaching to, and clicking on, one of two response areas, left or right side of the display, within 2000 ms (Fig. 2). The rectangular response areas, covering the leftmost and rightmost 23% of the display, were indicated by two vertical lines along the screen sides (x coordinates: 440 and 1480 pixels, respectively; see, Fig. 2). For half of the participants, edible was attributed to the left response area and non-edible to the right. For the other half, it was reversed. Response deadline was 2000 ms, after which the subject missed the trial. The deadline was introduced to create time pressure. This and similar methodological practices to encourage early movement initiation are used commonly in mouse-tracking studies^26,27^. In our particular protocol, this deadline had been established after previous pilots, and rendered average performance below ceiling but within the pre-set subject inclusion criteria (< 75%). As it will become clear later, the trial time imposed could be (and was) used up in different ways depending on the available information at the beginning of the trial (see “Movement onset latency analysis” in the Results section). Each trial took the whole 2000 ms, independently of the response time, to ensure that the duration of the session was fixed. After a trial ended, the participant needed to move the cursor back to the bottom-centre home location for the next trial to begin. The inter-trial interval was 2000 ms, which also served as a fixation screen. Trials from all three conditions (NB, LB, HB) were interleaved randomly throughout the experiment. Hence, for efficient responding, participants could not fall back on a pre-defined strategy based on visibility prior to the start of the trial.

Because the response areas covered both lateral sides of the display, the decision movement could vary in terms of the vertical extent of the trajectory, including direct horizontal movements from the home location to the response area. As said earlier, in the blurred image (LB and HB) conditions, the image blur decreased as the mouse moved upward. Therefore, when the image did not contain sufficient information, the participant needed to move in the vertical direction in order to gather evidence. Because of the response deadline (2000 ms), moving upward had a cost (i.e., took time off the available response time). Therefore, moving upward is not an optimal strategy if it is not necessary to sample evidence.

### Data analysis

**Figure 3:**
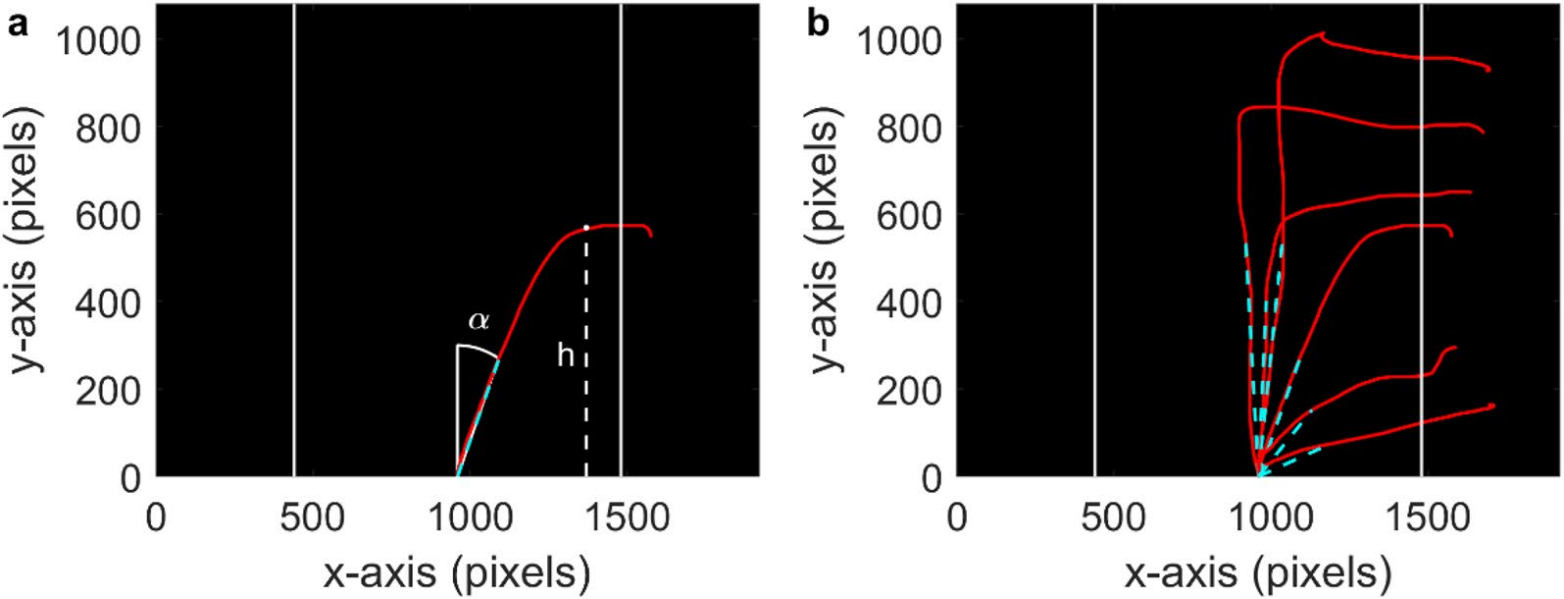
(**a**) An example of one mouse trajectory (red line) on the experimental display. Response areas are indicated to the participants by the solid vertical lines on the left and right sides. The white dashed line indicates the height *h* of the trajectory. The cyan dashed line that joints the origin with the point of the trajectory that lies at one third of its total length serves to calculate the initial angle α of the trajectory with respect to vertical. Positive angles are defined to be in the direction of the correct target, whose location could occur randomly on either side. (**b**) Examples of trajectories for several individual trials, with the same conventions described in a.

In our task, characterizing information sampling and response components of the subjects’ action boils down to the analysis of heights and angles of the response trajectories (some example trajectories are shown in Fig. 3). Firstly, we inspected the trajectory height, denoted *h*, which was calculated by measuring the vertical distance (in pixels) between the starting point and the highest point of the trajectory (Fig. 3a). Second, we analysed the initial angle of trajectories, denoted α, which was defined as the angle described by an imaginary straight line connecting the starting point with the point at one-third of the length of the trajectory (cyan dashed line in Fig. 3a), with respect to the vertical midline (0º). It is important to note that, although correct targets were randomly assigned left or right sides during the task, for analyses we realigned the correct choice to positive angles. Henceforth, positive angles indicate the direction of the correct choice, and negative angles that of the incorrect choice. Despite we excluded incorrect trials from the analysis, negative angles are possible at initial stages in the trajectory of correct trials.

We preregistered this study and we first report the analyses that were planned prior to data collection (see, https://osf.io/3ysah/). We also performed follow-up analyses that have been decided after the pre-registration process, as these reveal important characteristics of the data. Throughout the results section we report statistical tests according to the frequentist approach (the analogous Bayesian analyses are reported in the Supplementary Table S1, as both analyses lead to the same conclusions). We excluded incorrect trials from the trajectory analyses, as is usual practice in order to extract decision-related effects from categorically similar responses^28–30^. On average, each participant had 110 correct trials (range 103–123) out of 126 total (overall mean accuracy > 87%). The mean number correct trials out of 42 per condition was 37.4 (sd = 3.6), 36.9 (sd = 2.7) and 36.2 (sd = 2.8) for NB, LB and HB conditions, respectively. This indicates that the increase in blur ended up with slightly lower accuracy rates. The mean response time of correct trials was 1107 ms (sd = 117), 1266 ms (sd = 135) and 1374 ms (sd = 142) for NB, LB and HB conditions, respectively. The increase in blur resulted in longer response times in addition to lower accuracy.

## Results

### Movement-dependent information sampling

**Figure 4:**
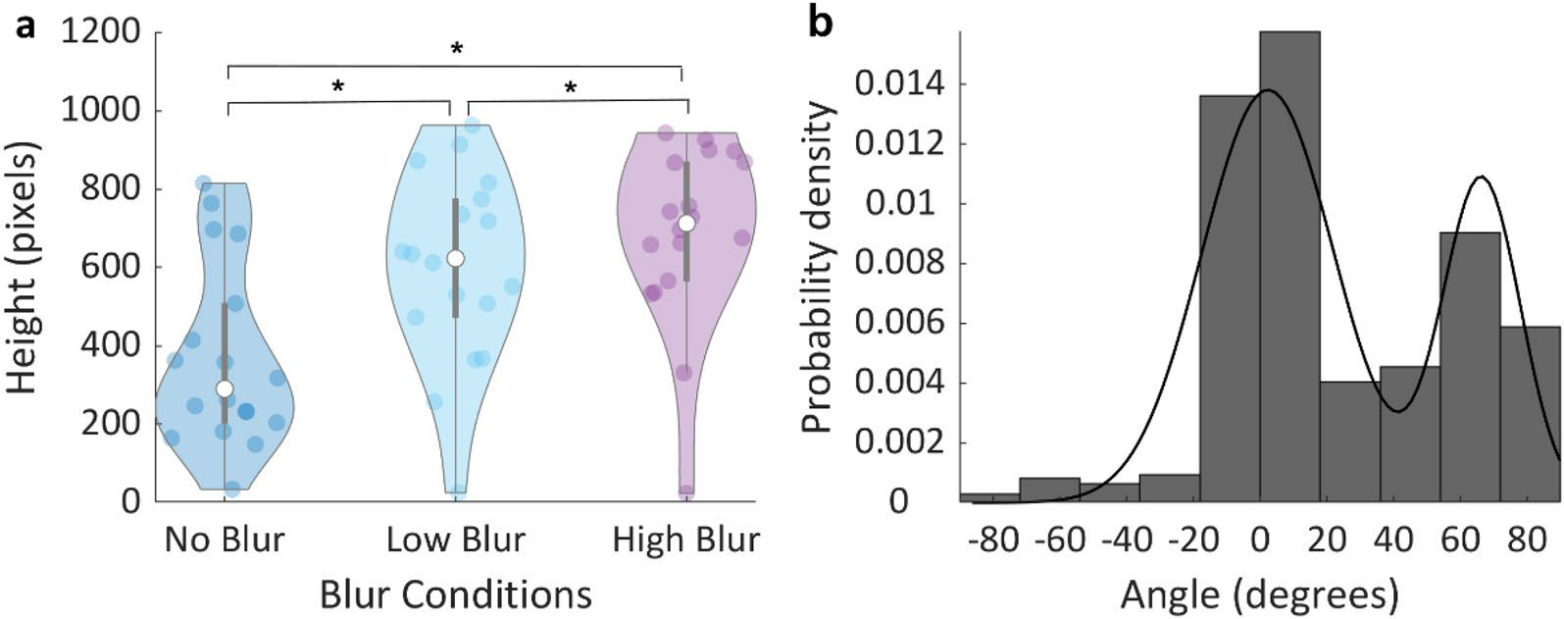
(**a**) Height of trial trajectories for NB, LB and HB conditions. Each colored dot represents individual means for the corresponding condition. White dots represent the group median for the condition and the grey lines represent the inter-quartile range. (**b**) Probability density of the initial angles of the trajectories across participants. The solid black line corresponds to the Gaussian mixture model (with 2 components) fit to the distribution (model with 2 components AIC = 19,105 < model with 1 component AIC = 19,753). Angle 0º corresponds to straight vertical upwards movement, i.e., with no horizontal component. Positive angles correspond to correct target direction.

If participants gather information as is needed, their trajectories should reach higher when the image is blurred. We therefore tested whether trajectories in blur trials reached higher than trajectories in the no blur trials. As can be seen in Fig. 4a, trajectories in the two blur conditions were higher than in the no blur condition, since information sampling was unnecessary in the latter (right tail paired-samples t-tests, t(17) = 6.53, p < 0.001, Cohen’s d = 1.54; t(17) = 7.03, p < 0.001, Cohen’s d = 1.66, for the comparison of NB with LB and HB, respectively). This result rules out the option that participants used a good-for-all strategy, by just moving up as soon as the trial started and then deciding which side to go. However, even in the NB conditions trajectories had a non-zero vertical component (mean = 368.3 pixels, sd = 231.9), possibly due to biophysical motor constraints. Another potential reason for non-zero height in NB condition is the random presentation of conditions in the experiment. Since in approximately two thirds of the trials gathering more information has an advantage, participants might have an anticipatory tendency to move upwards. To eliminate the height differences that are present in the trajectories but unrelated to information gathering, we subtracted the average height in NB condition from LB and HB trajectory heights in each individual’s data and continued the analysis with these normalized values. The results showed that trajectories in HB trials were about 27% higher than in LB trials (mean = 315.7, sd = 190.4, vs 229.3, sd = 148.8, respectively; right tail paired-samples t-test, t(17) = 5.39, p < 0.001, Cohen’s d = 1.27).

### Interplay between decision and action

#### Bimodality of trajectories

A central prediction of ECTs is that movements should reflect the decision-making process throughout, such that the trajectories should show early on a bias towards the finally chosen target. We tested this prediction by studying the initial angles of the trajectories (Fig. 4b). However, for this analysis we decided to include only those trials for which sampling had occurred, instead of mixing in trials with and without sampling behaviour. This was motivated by the fact that the distribution of angles was clearly bimodal (Hartigan’s Dip Test^31^, p-value < 0.001; Gaussian mixture model better fit with 2 components, Akaike Information Criterion (AIC) = 19,105 than the model with 1 component, AIC = 19,753). A central lobe of the distribution peaked at an angle 2.3º (that is, close to vertical, which was arbitrarily defined to be 0º), and a lateral lobe peaked at 66.4º (positive angles correspond to directions to the correct target, with 90º being a perfectly straight trajectory). The separation between the two lobes of the bimodal distribution was therefore 43.52º. Detecting subtypes of trajectories is fundamental to avoid averaging trials that are different in terms of the underlying cognitive modes^32,33^. Please, note that in our case averaging these two types of trajectories could end up rendering an average trajectory between sampling and non-sampling that is unrepresentative of the majority of the responses, which are of one or the other kind. Therefore, this bimodality and the cut-off point allowed us to classify trajectories as sampling or non-sampling, depending on whether the initial angle is closer to the central or the lateral peak of the bimodal distribution, respectively. Apart from the bimodality at group level, we confirmed significant bimodality in the distribution of trajectory angles for each subject individually (see Figure S1) for 9 out of 18 subjects. This means that early on in a trial, there is a fast sub-decision regarding the sampling or non-sampling strategy. This is further supported by the movement onset latency results, below.

The presence of two types of trajectories can be observed in each blur condition separately (Figure S2). As one would expect, there is a large fraction of non-sampling trajectories in the NB condition (corresponding to the lateral lobe of the bimodal distribution; q = 0.62, X^2^(1, N = 635) = 39.86, p < 0.001), though perhaps surprisingly in the HB condition there was a fraction of non-sampling trajectories (q = 0.14 binomial test p < 0.05). The presence of sampling and non-sampling trajectories across all blur conditions suggests that participants made an initial choice about whether or not to gather information. This is supported by an analysis that showed that trajectories classified as non-sampling had a much smaller height than sampling trajectories (right tail two-sample t-test, t(17) = 11.9, p < 0.001). Thus, non-sampling trajectories simply reflect a direct movement towards the chosen target that emanates from an initial decision, with little information gathering or ongoing decision-process throughout.

#### Movement onset latency analysis

We estimated the latency of movement onset as the time between trial onset and the initial movement of the mouse. The analysis showed that mean latency in non-sampling trials was longer (mean = 435 ms, sd = 125 ms) compared to sampling trials (mean = 329 ms, sd = 77 ms; right tail two-sample t-test, t(17) = − 4.4 , p < 0.001, Cohen’s d = − 1.04). This means that when the subjects exhibited a non-sampling strategy, they generally did so after waiting for longer at the initial location. This adds support to the interpretation that there is an initial sub-decision about whether to sample information or not, happening early in the trial, based on the available information about the target.

#### Speed of movement analysis

We estimated the average speed of trajectories in each condition. The mean speed was higher in HB (mean = 30.2 cm/s, sd = 6.9) and LB (mean = 28.8 cm/s, sd = 7.6) compared to NB (mean = 23 0.9 cm/s, sd = 7.5) condition. We conducted repeated measures ANOVA to see the effect of blur on movement speed. The results showed that movement speed was significantly modulated by blur (F(2,34) = 37.2, p < 0.001, η^2^ = 0.68).

#### Angle analysis of sampling trajectories

Thus, given the initial sub-decision and the ensuing existence of two different types of trajectories, a direct test of the prediction of ECT requires examining the sampling trajectories alone. These trajectories correspond to the central peak of the distribution in Fig. 4b. As the initial angles of these trials are close to zero (vertical), trajectories mostly depart vertically from the home position with the aim of gathering information to guide the final choice. However, a key finding is that in addition to the prominent vertical component, the initial steps of the trajectory were already biased towards the chosen target, as the initial angle was significantly larger than zero in both LB and HB conditions (right tail one sample t-tests, t(16) = 4.58, p < 0.001 and t(16) = 3.41, p = 0.002, respectively). This result strongly supports the notion that the decision process transpires into the movement even whilst participants are actively sampling information.

**Figure 5:**
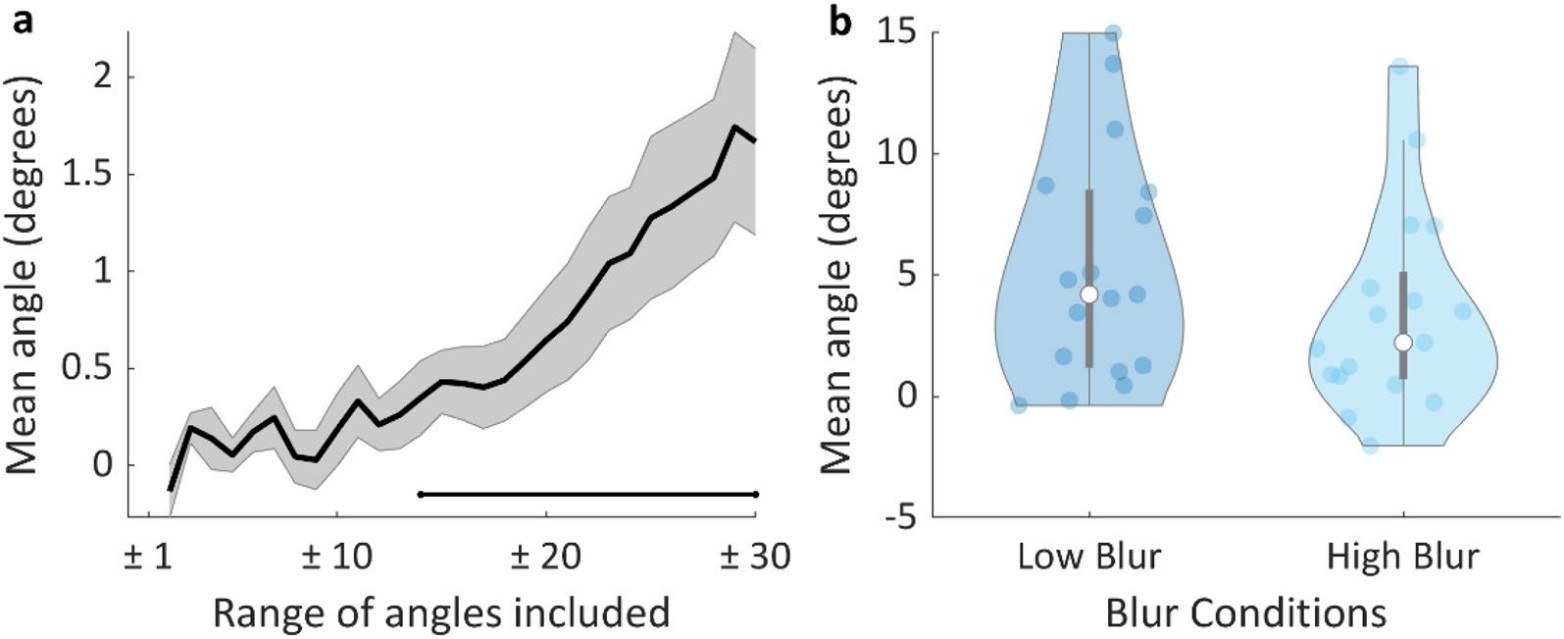
(**a**) Mean initial trajectory angle for all blur trajectories (pooled LB and HB data), along incremental ranges of angles symmetric around zero. The solid black line corresponds to the inter-individual mean (the grey area represents s.e.m.). The black horizontal line represents significance (right tail t-test, p < 0.05) against the hypothesis that the mean angle is not larger than zero. (**b**) Initial angle of trial trajectories for LB and HB conditions. The coloured dots represent each participant’s mean value for the corresponding condition. The white dots represent the median for each condition and the grey lines illustrate the inter-quartile range.

One might argue that some trials in the analysis above might have been misclassified (as non-sampling, instead of sampling trials), given the partial overlap of the two lobes of the bimodal distribution of angles. This could introduce some biases towards positive angles. To control for this possible confound we used a more data-driven analysis limited to LB and HB trials only (in which participants are, for the most part, in need to sample information), that does not rely on trial classification. In this analysis we calculated average angle in incremental ranges of angles (symmetric around 0º) from ± 1º to ± 30º, in steps of one degree (Fig. 5a). We found that the average angle was significantly larger than zero in all the ranges larger than ± 14º (right tail t-tests, p < 0.05, see Fig. 5a). Angles in the range ± 14º and ± 20º are well inside the central peak of the bimodal distribution, as described above, and therefore can be independently classified as sampling trajectories (trajectories with such small initial angle very unlikely correspond to trials where the decision maker already made a choice about where to move). In sum, this new analysis reveals that trajectories whose initial angles lie within a small range of angles symmetrical around zero already show a significant bias towards the chosen target. This result supports, once more, the notion that the ongoing decision-making process transpires into the movement well before all the information necessary to solve the task has been gathered.

Although we did find significant deviations in the initial angle of blur trials (HB, LB) we did observe only marginal evidence that the angle deviation was larger in LB (mean = 5.28º, sd = 4.75) than in HB (mean = 3.42º, sd = 4.13) conditions (Fig. 5b; right tail paired-samples t-test, t(16) = 1.66, p = 0.058, Cohen’s d = 0.4).

### Converging evidence from angle and height information

**Figure 6:**
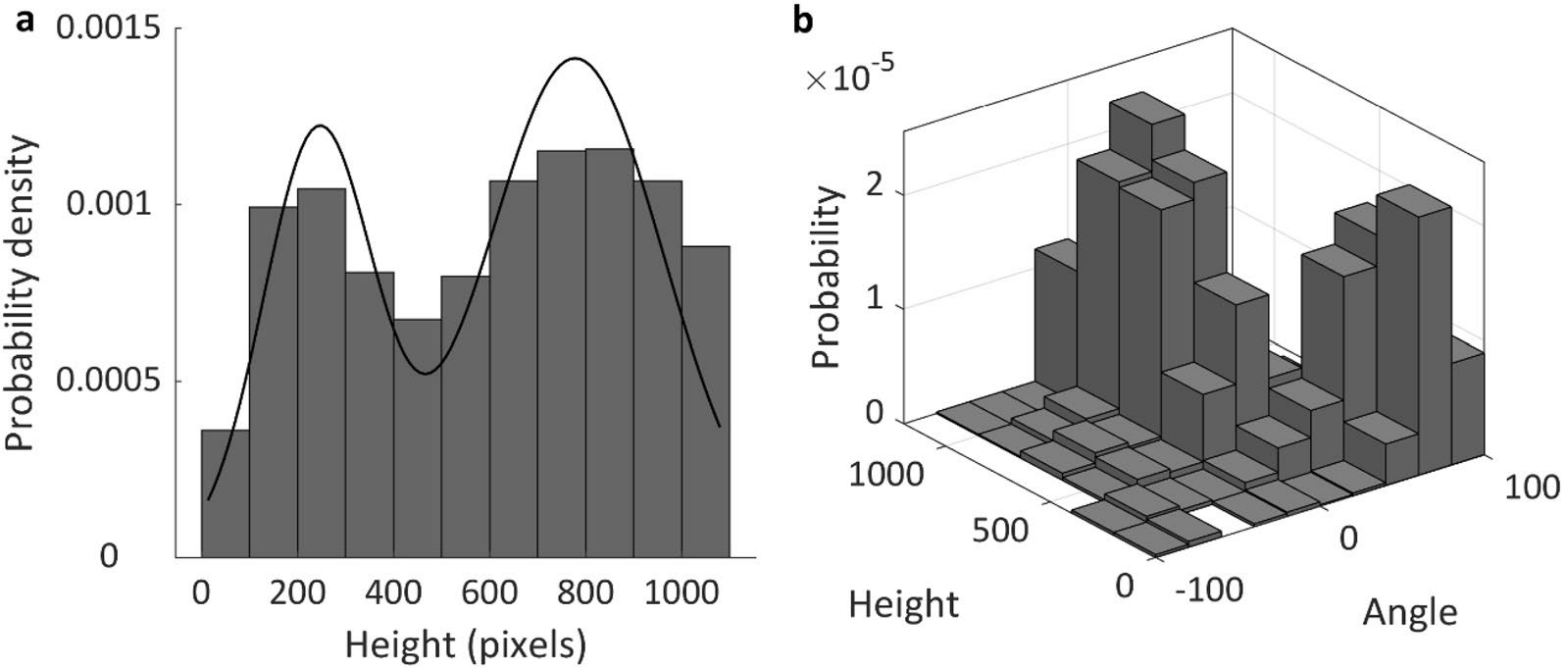
(**a**) Probability density of the heights of the trajectories across participants. The solid black line corresponds to the Gaussian mixture model with 2 components fit to the distribution (better fit in the model with 2 components, AIC = 26,439 lower than the model with 1 component, AIC = 26,874). (**b**) Probability density of the heights and angles of the trajectories across participants.

Initially we had decided to classify sampling and non-sampling trials based on initial angle of the trajectories. However, if our hypothesis is correct, a similar classification should apply to the heights of the trajectories. This is because sampling trajectories are expected to reach higher than non-sampling trajectories, as the latter correspond to ballistic movements to the target without much ongoing deliberations and thus are expected to reach vertically much lower. What is more, if trajectories are truly separable into sampling and non-sampling, then it should be the case that in their heights should also be distributed in a bimodal way, and height and angle should be correlated. Consistent with this prediction, we found that heights were distributed in bimodally (Fig. 6a) across conditions and participants (Fig. 6a; Hartigan’s Dip Test, p < 0.05; see Figure S3 for each blur condition). These results in turn suggest that it should be possible to classify trajectories as sampling and non-sampling based on the bimodality in heights, and that this classification should be largely consistent with the one derived above from the angle analyses. In line with this, classification based on height and classification based on angle were highly correlated (Pearson’s correlation, r = 0.76) and clustered trials in two clear categories (Fig. 6b).

Similar to the main angle analysis reported in Sect. 3.2 (where trial classification was based on angle), we analysed angle again but this time using trial classification based on height. We found that the angles in sampling trials, both the LB and the HB conditions, were significantly larger than zero (right tail one sample t-test, t(16) = 3.7, p < 0.001, Cohen’s d = 0.9 and t(16) = 2.05, p = 0.029, d = 0.5, respectively). This outcome corroborates the conclusions of our main analysis and shows that this finding generalizes regardless of the classification variable used.

### Generalization of the results along the trajectory

**Figure 7:**
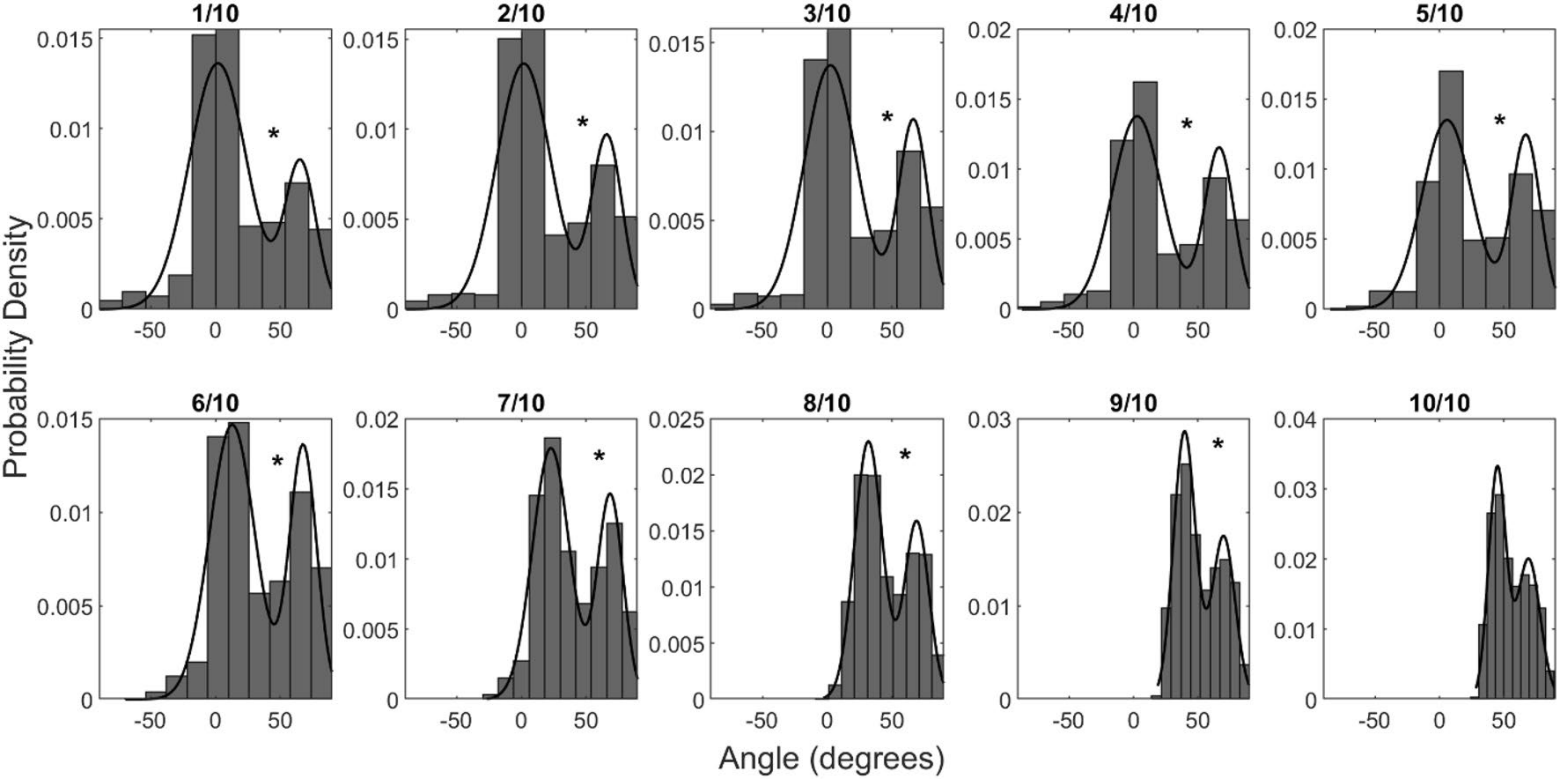
Probability density of angles calculated at 1/10th to 10/10th of trajectory length. The solid black lines correspond to the Gaussian mixture model with 2 components fit to the distribution. The asterisks indicate the significance of Hartigan’s dip test for bimodality (p < 0.05).

In the main analysis, we estimated angles at one third of the trajectory, as we wanted to capture the initial moments of the response movement. However, the criterion to compute angles at one-third of the trajectory is somehow arbitrary. As a check regarding the reliability of this result, and the validity of the criterion used, we decided to compute the angles along the whole trajectory at 10 equidistant points, from 1/10th to 10/10th of the trajectory length. Then we checked the distribution of angles at each of these trajectory points. We found significant bimodality of angle distributions in all except the last trajectory point (Hartigan’s Dip test, p < 0.05). This generalizes the bimodality of trajectories beyond the one particular point used in the main analysis. As can be seen in Fig. 7, the distribution of angles from 1/10th to 5/10th of trajectory shows an earlier peak closer to 0º which means that a portion of trials classified as sampling are still in a phase of upward movement. Logically, at later stages the trajectories show diversion towards the final choice. Therefore, we are safe to interpret our main results obtained from angles calculated at the 1/3rd of the trajectory length as it is early enough to check if decision transpires into the movement during active sampling.

### Angle and height analyses including error trials

It is common practice to use only correct trials in trajectory analyses^28–30^, because the aetiology of errors is varied and difficult to trace. However, one could argue that removing error trials might have biased the outcomes toward positive angles. In order to ensure that the results we found were not due to biases induced by the exclusion of error trials, we repeated the height and angle analysis, this time including error trials along with the correct trials. We found that NB trials (mean = 393.6, sd = 208) had significantly lower height than LB (mean = 636.6, sd = 185.7) and HB (mean = 726.8, sd = 150.8) conditions (right tail paired-samples t-tests, t(17) = 7.6, p < 0.001 and t(17) = 8.1, p < 0.001, respectively). As in the main Results Sect. 3.2, we subtracted the NB average height from LB and HB and compared them. The result of the right tail paired-samples t-test showed that HB trajectories were significantly higher than LB trajectories (t(17) = 5.2, p < 0.001), confirming the main results conducted only on correct responses. Similarly, we assessed the angle of sampling trajectories in LB (mean = 4.6, sd = 4.2) and HB (mean = 2.1, sd = 3.9) and found that both were significantly above 0 (right tail one sample t-tests, t(17) = 0.4.7, p < 0.001 and t(17) = 2.3, p < 0.05, respectively). We conducted right tail paired-sample t-test, to test if LB had larger angles than HB. The results showed that LB had significantly larger angles than HB (t(17) = 2.5, p < 0.01). Thus, we can conclude that the results we reported were not biased due to exclusion of error trials. As we see that the direction and significance of the effects did not change when the analysis were repeated with correct and incorrect trials altogether.

## Discussion

Many studies in the past have challenged the classical view of decision-making and cognition which assumes a temporal and functional separation between decision and action systems^1,2^. The newer view is that natural choice actions in humans and other animals involve movement patterns that reflect, in part, the ongoing decision process. As a result, movement trajectory analyses in continuous control tasks have been increasingly used to trace the underlying decision dynamics. The outcome of the present study clearly sides with this framework, showing that it is possible to trace decision dynamics from the ongoing choice action^34–36^. However, the majority of the tasks used in previous studies did not contemplate decision-making scenarios where actions are also required to sample information. This scenario characterises choice in many natural environments, such as getting closer to an object to decide whether it is nutritious food or else should be avoided. To fill this gap in the literature, we tested whether the outcome of decision processes pervades sampling actions.

As mentioned in the introduction, parallel processing of decision-making and action control processes is an important principle. However, the nature of the interaction between the two is still under debate, given that a strictly parallel view might be insufficient to account for the full range of decision and action interactions. For instance, Lepora and Pezzulo^20^ have put forward the ‘embodied choice’ framework, that accommodates richer interactions between action and decision through action-dependent information gain, compared to the parallel account. However, the experimental tasks they have used to illustrate their predictions lacked the active sampling component, which leaves one main prediction of the theory still unresolved. The findings of the current study support the ‘embodied choice’ theory by showing that the interaction between decision and action can be revealed, and traced in the decision responses, under ecological scenarios that incorporate the active sampling constrain. If this were not the case, we would have observed a temporally separated sampling and responding characteristics in the movement trajectory without any angular deviation during sampling, in early parts of the trajectories. In fact, that non-sampling trajectories observed in our data revealed a kind of serial decision-making pattern which consists of a longer stationary period followed by a shorter movement (Results section, movement onset analysis). However, sampling trajectories were characterized by moving earlier (shorter stationary period) followed by a longer movement directed at sampling which is biased by the decision process. Thus, rather than claiming that all decisions are fully parallel and continuous, our preferred interpretation is that, even if there are certain stages in the decision process, some of them allow for continuous interaction of action and decision status.

One central feature of the task used in the present study is that participants must trade off information (image de-blurring) for energetic efficiency (moving up, hence orthogonal to the choice goal). This is because motor execution involves expenditure of energy, thus incurring effort-related costs. Motor cost and physical effort have started to be studied in relation to decision-making^7,15^. For instance, Cos and colleagues^37^ have shown that effort and biomechanics of a task influence the decision dynamics starting at early stages. It is likely that physical effort influences the decision dynamics due to the strong interactions between action and decision. In our experiment, each blur condition had a different cost/information structure. Although, it is not easy to quantify exactly how this effort-to-information ratio impacted our results (due to the use of real images instead of parametric stimuli), it is still safe to say that the effort associated to information sampling altered the decision-making process, rendering differences in choice trajectories. The analyses showing an inverse relationship between image visibility and trajectory height clearly support this.

The main result to emerge from this study, however, was based on the deviations and curvatures in choice trajectories. Please note that this is only superficially similar to other mouse-tracking studies^38–40^. A common task characteristic our current study shares with this previous work is the urgency of responding^26,41^. Via imposing time pressure, participants are encouraged to execute decision and action in the same time window as it is more optimal for a successful response than staying stationary to make a decision and then move to report it. However, the fundamental feature of our experiment compared to others in the decision-making literature is the presence of a functional link between information and movement. In those previous works, the subject planned and performed actions to report the choice response, therefore effectively allowing to study interactions between decision process and response plan only in one direction (as shown in Fig. 1a). In contrast, the task we developed here involves, and makes it possible to study, both response and sampling plans and their mutual interplay (Fig. 1b). Another way to put it is that most of the previous studies so far have considered only tasks equivalent to the ‘no blur’ condition of our study. Hence, one of the main goals here was to compare the trajectories between different sampling conditions as a function of movement-to-information ratio. First, the results obtained conclusively support the prediction that the decision process pervades information sampling movements in various ways. Information sampling trajectories deviated toward one of the choices (the correct one, on average) very early on. We confirmed this both in low blur and high blur conditions, using only trials classified as sampling trials. A second expectation by hypothesis was that, if the sampling component was stronger in high versus low blur conditions, then one would assume that the decision component will be more pronounced in the trajectories of low blur trials than in those of high blur trials, especially at early stages. This is because the need for information in high blur trials is stronger. Angular differences between low and high blur conditions calculated according to the planned analysis (at 1/3th of trajectories) were in the expected direction, but reached only a marginally significant effect. This borderline result may be due to the fact that the two conditions were not sufficiently different in terms of costs of sampling movement (effort-to-information ratio). This cost depended directly on the blur function, which was chosen arbitrarily. Indeed, subsequent analyses where angle was calculated at different stages throughout trajectories, or when angular deviation was calculated in incremental steps from movement origin, revealed robust significant differences in the same, predicted direction. This variability reflects the importance of the task mechanics to the study of sensorimotor interactions in a decision-making setting^41^. Variants of active sampling decision-making tasks, including variations of the information cost function, should shed more light on the full range of embodied decisions under naturalistic constrains.

We argue that the proposed interactions between action and decision revealed by our data rely on the incorporation of sampling and responding actions in the task structure (as illustrated in Fig. 1b). We note that the tasks that include movement-agnostic stimulus, often used in the literature (and illustrated in Fig. 1a), are a special instance of the more general case modelled in Fig. 1b: one in which the arrows to and from “sampling plan” have zero weight. This is also the case of non-sampling trajectories that we observed in our study. Yet, our experimental setup is not intended as a general model for all action-decision possibilities that humans and animals are capable of. We rather claim that embodied decisions are the manifestation of the flexibility of the decision process^42^. In many natural and ecological situations, like the one modelled here, decisions have to be carried out as ECTs predict—with a strong interaction coupling with action processes. Nevertheless, there are also abstract and higher-level decisions which may comply with serial accounts of decision-making, especially in humans given their more sophisticated planning strategies. In line with a ‘phylogenetic refinement’ view, fully abstract cognitive operations are evolutionarily more recent, whereas rich cycles of action and decision are prevalent from very basic animals to complex mammals^43^. In the human context, depending on the task, the biomechanical characteristics and previous experience, we may observe response patterns ranging from a pure abstract and covert decision-making process that precedes any action, to a fully embodied and interactive one such as the one seen here. For instance, a novice driver may find herself thinking step-by-step about all of the driving actions before executing them, however as practice accumulates, she may decide and move at the same time with ease. Therefore, we are aware of the vast complexity underlying the interaction between decision and motor action^44^. Previous studies have succeeded in revealing the impact of decisions on choice actions in situations where actions do not contribute information. Our study provides one step forward in understanding these interactions under the new constrain of action-dependent information sampling. What we have shown is that when the task dynamics imposes this type of ecological constraint, action for sampling and choice action have interactions with the decision process and with each other.

Despite the novelty of the present study, it has covered only a subset of situations and some areas of the decision-action process remain uncharted. For instance, in this study we used orthogonal vertical and horizontal movement components to observe sampling and decision respectively. Yet, the weights of these movement axes are not equal, considering the display dimensions and the difficulty of equating a level of information gained with a unit of sampling movement and a level of decision criterion with a unit of response movement. In the future, different approaches such as reward structures and/or stimulus that allow parametric information gain can be utilized to answer more specific questions about action and decision interaction. Besides the task design aspects, we are not oblivious to the fact that the present group patterns on which we have based our conclusions contain important individual differences. These individual patterns may reveal fundamentally different strategies in the trade-off between information sampling and decision. Uncovering the hidden dynamics behind them will be key to characterize embodied decisions. Lastly, in this study we have focused our analysis on the correct trials and therefore designed a task with a ceiling level performance. In decision-making field, error responses are crucial to understand the underlying mechanisms. After establishing the main principles of the ECTs, we expect to see studies delving on to error behaviours and enriching our understanding of embodied decisions.

To summarize, the present study provides a demonstration of interactions between action to sample information, action to respond, and decision process with a novel mouse-tracking task. Our results show that decision outcomes feed into movement trajectory during information sampling movements which, in turn, accrue decision-relevant information. This is a support for the embodied theories in decision-making with a task that allows to inspect rich action-dependent sampling mechanisms.

## Supplementary Information

Supplementary Information.
