# Supplementary material for: The dynamics of decision-making and action during active sampling
Source: Sci Rep. 2021 Nov 29;11:23067. doi: 10.1038/s41598-021-02595-3 (PMC8630054; doi:10.1038/s41598-021-02595-3)
Supplement: Supplementary file 1 — Supplementary Information. [file 41598_2021_2595_MOESM1_ESM.docx]

**Supplementary Figures**


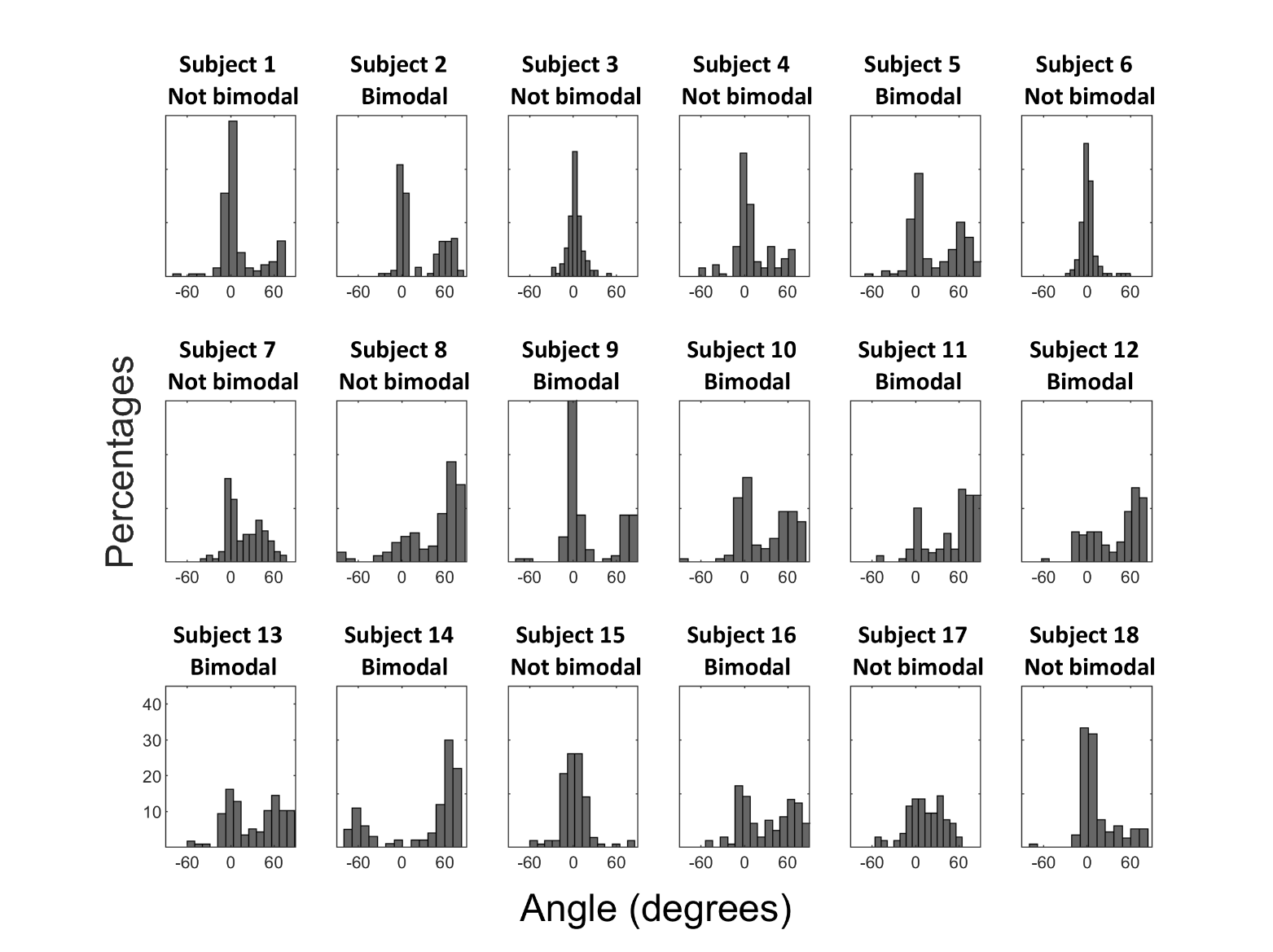


**Figure S1.** Distribution of angles for each individual subject.

**
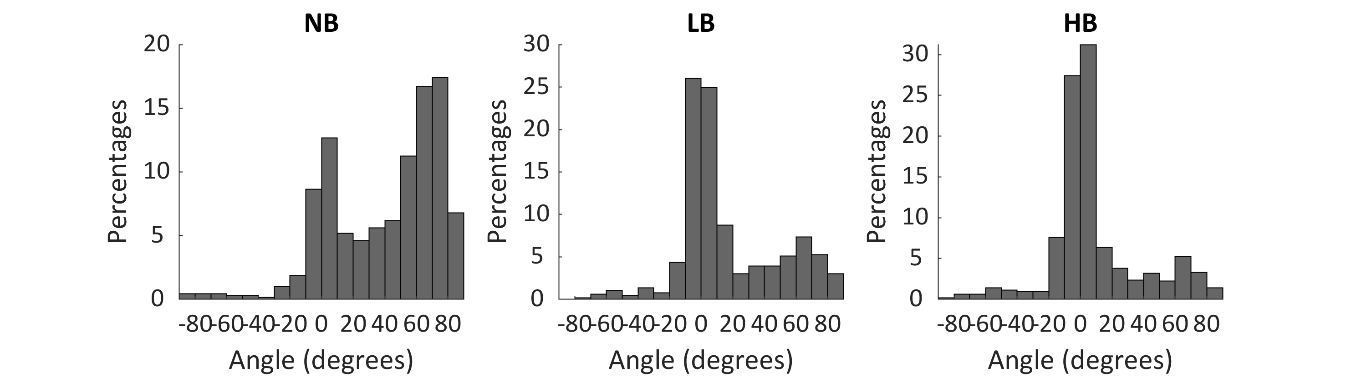
**

**Figure S2.**  Distribution of trajectory angles for each blur condition


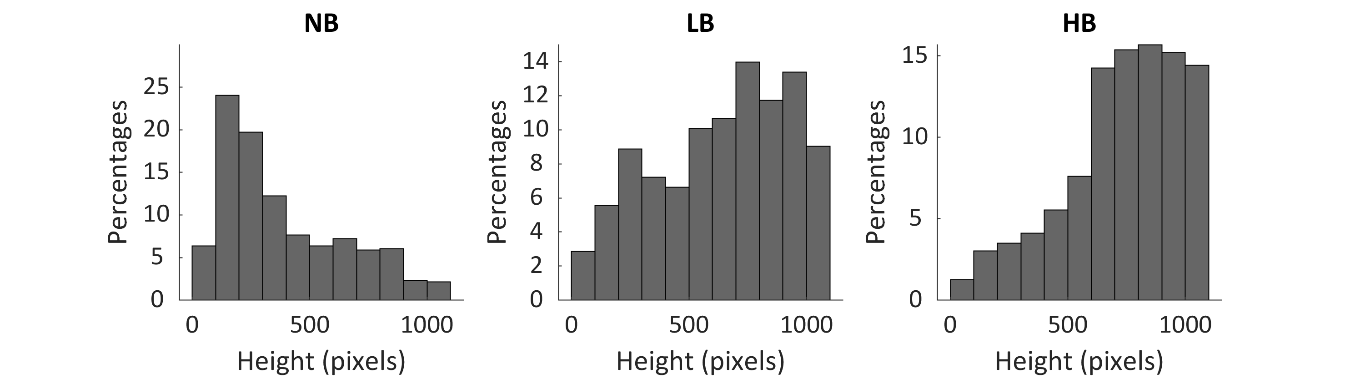


**Figure S3.** Distribution of trajectory heights across participants for each blur condition.

**Supplementary Table**

|  | Page | Analysis Variable | H1 | Bayes Factor | Error | Median Effect Size | 95% CI | Frequentist  p-values |
| --- | --- | --- | --- | --- | --- | --- | --- | --- |
| 1 | 7 | Height (all trials) | HB > LB | 1067.6 | < 0.001 | 1.17 | [0.54, 1.79] | <0.001 |
| 2 | 9 | Angle (classification based on angle) | LB > 0 | 209.53 | <0.001 | 1.01 | [0.41, 1.63] | <0.001 |
| 3 | 9 | Angle (classification based on angle) | HB > 0 | 25.27 | <0.001 | 0.73 | [0.21, 1.29] | =0.002 |
| 4 | 10 | Angle (classification based on angle) | LB > HB | 1.45 | 0.004 | 0.37 | [0.04, 1.12] | =0.058 |
| 5 | 11 | Angle (classification based on height) | LB > 0 | 44.02 | <0.001 | 0.8 | [0.26, 1.38] | <0.001 |
| 6 | 11 | Angle (classification based on height) | HB > 0 | 2.56 | 0.002 | 0.44 | [0.06, 0.93] | =0.029 |

**Table S1.** Bayesian counterparts of the t-tests that have been reported in the Results section. The analyses are ranked in the order of appearing in text.
